# Upregulation of Translationally Controlled Tumor Protein Is Associated With Cervical Cancer Progression

**DOI:** 10.3389/fmolb.2021.686718

**Published:** 2021-09-13

**Authors:** Xiaoyu Zhu, Ji Ren, Dianqin Xu, Di Cheng, Wei Wang, Jie Ren, Ziwen Xiao, Hongmei Jiang, Yan Ding, Yujie Tan

**Affiliations:** ^1^ School of Laboratory Medicine, Guizhou Medical University, Guiyang, China; ^2^ Affiliated Hospital of Guizhou Medical University, Guiyang, China; ^3^ Affiliated Oncology Hospital of Guizhou Medical University, Guiyang, China; ^4^ Taihe Hospital, Affiliated to Hubei Medical University, Shiyan, China

**Keywords:** cervical cancer, TPT1, PI3K/Akt/mTOR pathway, diagnosis, biometrics

## Abstract

Outside a few affluent countries with adequate vaccination and screening coverage, cervical cancer remains the leading cause of cancer-related deaths in women in many countries. Currently, a major problem is that a substantial proportion of patients are already at an advanced cancer stage when diagnosed. There is increasing evidence that indicates the involvement of translationally controlled tumor protein 1 (TPT1) overexpression in cancer development, but little is known about its implication in cervical cancer. We assessed the levels of TPT1 in surgical tissue and sera of patients with cervicitis, cervical intraepithelial neoplasia III, and cervical cancer, as well as in normal and cancerous cervical cell lines. Gene sets, pathways, and functional protein interactions associated with TPT1 were identified using the TCGA data cohort of cervical cancer. We found that the TPT1 expression was significantly increased in cervical cancer tissue compared to all nonmalignant cervical tissues, including samples of cervicitis, cervical intraepithelial neoplasia III, and normal controls. Serum level of TPT1 was also increased in cervical cancer patients compared to healthy subjects. Furthermore, elevated TPT1 expression was significantly correlated with lymph node metastasis and a low differentiation degree of the cancer. In the cancerous tissues and cell lines, selective markers of PI3K/AKT/mTOR pathway over-activation, apoptosis repression, and EMT were detected, and their interaction with TPT1 was supported by biometrics analyses. Our results, for the first time, demonstrate a strong correlation of upregulated TPT1 expression with cervical cancer progression, suggesting that TPT1 might provide a potential biomarker for cervical cancer progression.

## Introduction

Translationally controlled tumor protein 1 (TPT1/TCTP, aka histamine-releasing factor HRF and fortilin) is a highly conserved protein abundantly present in all eukaryotic organisms (Li et al., 2001) and is involved in almost all fundamental biological processes underpinning growth, stress response, and survival (Bommer and Telerman, 2020). In addition to its intracellular functions, TPT1 can be secreted to stimulate histamine production of basophils to elicit allergic reactions and inflammation (Kawakami et al., 2019).

TPT1 is frequently elevated in cancers, leading to suppression of apoptosis, promotion of metastasis, and resistance to anticancer therapy (Koziol and Gurdon, 2012; Lee et al., 2020). TPT1 overexpression in circulation or tissues of patients has been documented in leukemia (Yağcı et al., 2013) and cancers in most main human organs such as the lung (Chen et al., 2013; Sun et al., 2019), liver (Chan et al., 2012; Lin et al., 2020), colon (Bommer et al., 2017), prostate (Kaarbø et al., 2013; Rocca et al., 2015), breast (Neuhäuser et al., 2019), etc. (Koziol and Gurdon, 2012; Ambrosio et al., 2015; Chen et al., 2015; Phanthaphol et al., 2017; Bommer and Telerman, 2020). The pro-survival roles of TPT1 manifest themselves in multiple and correlated facets of tumorigenicity and malignant transformation, including genome stability (Li et al., 2017), cell cycle, apoptosis machinery (Nagano-Ito and Ichikawa, 2012), mitotic/meiotic progression (Bommer and Telerman, 2020), PI3K/AKT/TOR signaling (Bommer et al., 2015), epithelial-to-mesenchymal transition (EMT) (Mishra et al., 2018), induction of pluripotent stemness (Amson et al., 2013), autophagy (Lee et al., 2020), and the reciprocal constraint between TPT1 and the p53-dependent tumor suppressor pathway (Amson et al., 2011).

Recently, inhibition of TPT1 to attain tumor reversion has provided a new approach for cancer therapy (Tuynder et al., 2002). The TPT1 gene was identified as the most differentially downregulated in revertant human leukemia cell U937 and breast cancer cells such as MCF7, compared with their malignant counterparts (Tuynder et al., 2002). Inhibition of TPT1 by anti-sense cDNA or siRNA *in vitro* or injection of antagonistic drugs, such as anti-histaminic compounds into tumor-bearing mice, suppressed the malignant phenotype of cancers of the breast, lung, and colon and melanoma (Tuynder et al., 2004).

However, hampered by the inherent difficulties of research resources, our knowledge of the association between TPT1 and human cervical carcinoma is largely absent. As per the global cancer statistics, cervical cancer is ranked as the fourth most common cancer in women (https://www.who.int/health-topics/cervical-cancer#tab = tab_1) (Das, 2021). Diagnosis and treatment of the preinvasive lesions at cervical intraepithelial neoplasia (CIN) stages would render the disease curable (Castle et al., 2017) and possess tremendous importance in the cervical cancer control agenda (Preti et al., 2020). Although the cervical cancer screening and prophylactic vaccination against HPV have achieved some success in prevention and early treatment in the most affluent countries, cervical cancer remains the leading cause of mobility and mortality for women in countries with inadequate health services and vaccination coverage (Bray et al., 2018; Beddoe, 2019; Canfell et al., 2020).

While no clinical evidence is available, a few works have used human papillomavirus (HPV)-infected cervical cancer cell lines to examine the role of TPT1 in cell survival. Forced TPT1 overexpression protects HeLa cells from cytotoxic drug-induced cell death, *via* interfering with the mitochondria-mediated apoptosis pathway (Jung et al., 2014). TPT1 expression is inducible by incubation of HeLa cells under stimulation of serum, which can be reversed by rapamycin treatment, implying that TPT1 is tightly regulated by the growth factor/PI3K/AKT/mTOR network (Bommer et al., 2015). In another study using a mouse cervical cancer model inoculated with HeLa cells, TPT1 protein expression increased in the advanced tumor but not in the stage of tumor initiation. The authors speculated that the TPT1 level is inversely related to the apoptotic activity (Hou and Zhai, 2015). However, the function of TPT1 in inflammation restriction *via* negatively regulating autophagy (Bae et al., 2017) may overrun its pro-proliferative action. TPT1 is identified as one of the most upregulated genes in SiHa cells treated with anti-inflammatory peptide Annexin A1 (*ANXA1*) and might have contributed to the inhibition of cell proliferation (Prates et al., 2015).

In the present study, by analyzing both tissue and serum TPT1 levels from clinical subjects and online databases, we attempt to clarify the relevance of TPT1 to cervical cancer development and the potential value of serum TPT1 as a facile indicator of cancer diagnosis and prognosis.

## Materials and Methods

### Patient Samples

Ethics approval for the study was obtained from the Human Ethics Committee of the Guizhou Medical University. A waiver of documentation of the written informed consent form of the subjects was issued by the Human Ethics Committee of the Guizhou Medical University, and oral informed consent was obtained from the patients.

Surgically resected cervical tissue samples were collected from patients treated in the Gynaecology Department at the Affiliated Hospital of Guizhou Medical University between January and September 2018. Information of samples used in different experiments, such as diagnosis, pathologic staging, and case number, is summarized in Tables 1, 2, 3. Samples were divided into four groups according to the pathological diagnosis, including cervical cancer *(n* = 36, age 31–70, mean age 48.50 ± 9.35), cervical intraepithelial neoplasia grade III (CINIII. *n* = 35, age 27–70, mean age 44.74 ± 11.23), cervicitis (*n* = 30, age 22–68, mean age 42.87 ± 10.56), and the control group (*n* = 30, age 30–54, mean age 45.13 ± 4.91). The control group included patients who underwent hysterectomy because of fibroid uteri but for whom the cervix tissues were confirmed to be normal by histopathological diagnosis postoperatively. In parallel, serum samples were collected from patients with cervical cancer (*n* = 40, age 26–69, mean age 47.55 ± 10.92), patients with CINIII (*n* = 40, age 23–60, mean age 42.68 ± 9.08), and healthy women (*n* = 40, age 31–56, mean age 45.18 ± 7.45). There was no statistically significant difference in age between these groups (*p* > 0.05). Another set of tissue specimens was obtained from cervical cancer patients (*n* = 20, age 31–68, mean age 48.85 ± 8.74) at the surgery and paired into cancerous and paracancerous groups. Cancer tissue and paracancerous tissue adjacent to and at the outside of the edge of cancer were dissected by the responsible surgeon and evaluated by tissue appearance, including color, texture, and hardness. All samples were confirmed through pathological examinations performed independently by two chief pathologists at the hospital.

**TABLE 1:** TPT1 staining positivity rate (%) in cervical tissues of different groups.

Group	Number of cases	Positive cases	Positive rate (%)	*p*-value
Control	30	2	6.7	N/A
Cervicitis	30	8	27	a
CINIII	35	16	46	a
Cervical cancer	36	25	69	a, b, c

a, b, c, *p <* 0.05 comparing to the control, cervicitis, and CINIII samples, respectively.

**TABLE 2:** Comparison of TPT1 content in serum of different groups of patients (means ± SD).

Group	Number of cases	TPT1 (pg/ml)	*p*-value
Normal	40	52.48 ± 15.01	N/A
CINIII	40	54.71 ± 15.89	ns
Cervical cancer	40	66.40 ± 30.98	0.028^a^

a *p <* 0.05; ns, not significant.

**TABLE 3:** Correlation between TPT1 expression level and patient clinicopathological features.

Variables	Number of cases	TPT1 expression		*p*-value
		High	Low	
Ages				
≤45	7	2	5	0.081
>45	13	9	4	
FIGO stage				
Ⅰ	12	4	8	0.199
Ⅱ	8	5	3	
Ⅲ	0	0	0	
Ⅳ	0	0	0	
Lymph node metastasis				
Positive	7	6	1	0.019^a^
Negative	13	4	9	
Differentiated degree				
Low	5	4	1	0.035^a^
Medium	15	4	11	
High	0	0	0	

a *p <* 0.05.

The study had the following inclusion criteria: 1) diagnosis of cervical cancer and CINIII were based on the Consensus Guidelines for the Management of Abnormal Cervical Cancer Screening Tests in China, combined with medical history, cytology, and pathology biopsy evidence, and 2) the patients had not received chemo-/radio-/hormone therapy or taken other medications prior to the hospital admission. Subjects were excluded if the patient had concurrent conditions of infectious disease, autoimmune disease, metastatic malignancy in the pelvic area, or cancers in other organs such as the breast, liver, kidney, and spleen or had received immunosuppressive therapy within a half year prior to the hospital admission.

### Cell Culture

The normal human cervical epithelial cell line HCerEpic (Beijing Beina Chuanglian Biotechnology Institute, China) and the human cervical carcinoma SiHa and HeLa cells (Nanjing Kebai Biotech Co., Ltd., China) were used. The cells were cultured in DMEM (Gibco, United States) supplemented with 10% FBS (Biyuntian Biotech Co., Ltd., China) and grown in an incubator at 37°C in 5% CO_2_ with 97% humidity. Cell medium was replenished every 2–3 days and passaged with 0.25% trypsin when the cell confluency reached 80–90%.

### Immunohistochemistry of TPT1 Protein Expression

The formalin-fixed, paraffin-embedded tissue samples were freshly sliced and subjected to immunohistochemistry. Tissue sections were sequentially deparaffinized, rehydrated, and treated in citric buffer for 3 min for antigen retrieval and 3% hydrogen peroxide for 30 min to block endogenous peroxide activity. After blocking with goat serum for 30 min and washing with PBS 3 times, sections were incubated with anti-human TPT1 primary antibody (1:1,000, ab133568, Abcam, United States) at 4°C overnight. Next, sections were similarly washed and incubated with HRP-conjugated goat-anti-rabbit immunoglobulin (PV-6001, Beijing Zhongshan Jinqiao Biotech Co., Ltd.) at room temperature for 2 h. After washing with PBS, the sections were developed in chromogen solution of 3′-3′-diaminobenzidine (DAB, Beijing Suolaibao Biotec Co., Ltd., China) for 2–3 min, followed by hematoxylin counterstaining, hydrochloric acid differentiation, bluing, and dehydration, and finally mounted for visual inspection under a microscope.

Slides were examined in five randomly selected high-resolution fields (400 x), and the staining score method that combines quantitatively counting the number of positively stained cells and qualitative evaluation of color intensity was adopted, using validated liver cancer slides as the positive control (Chan et al., 2012) and PBS to replace the primary antibody as the negative control. For the color intensity score, slides were assigned a score of 0 (unstained), 1 (weak), 2 (moderate), and 3 (strong). The staining area score was given for the percentage of positive cell count in the investigated areas: 0 (0%), 1 (1–25%), 2 (26–50%), 3 (50–75%), and 4 (>75%). The final immunoreactivity score (IRS) was presented as the staining intensity score × staining area score. An IRS of “0–4” was defined as negative, and an IRS of “5–12” was defined as positive (Chen et al., 2017).

### Measurement of Serum TPT1 Using Enzyme-Linked Immunosorbent Assay

A commercially available ELISA kit (CSB-EL024134HU, Wuhan Huamei Biotech Co., Ltd., China) was used for serum TPT1 detection, following the manufacturer’s instruction. Briefly, 100 μl diluents of test samples and protein standards with concentrations ranging from 0 to 800 pg/ml were added into the 96-well plate provided in the kit. After incubation at 37°C for 2 h, 100 μl biotinylated antibody was added to each well and incubated at 37°C for 1 h. Then the plate was subjected to a thorough wash and spin-drying before 100 μl of horseradish peroxidase-labeled avidin was added and the plate was incubated at 37°C for 1 h. After another round of washes and spin-drying, 90 μl of the substrate solution was added, and the plate was kept from light at 37°C for 15–30 min. Upon termination of the reaction with 50 μl stop solution, the optical density (OD) at 450 nm was measured using a microplate reader. The readings of OD were normalized with a background value obtained from blank sample wells, and the sample concentration was calculated according to the standard curve.

### Western Blot

50–80 mg cervical tissues were cut into small pieces and ground in liquid nitrogen to extract cell lysates for protein assessment. HCerEpic, SiHa, and HeLa cells in the logarithmic growth phase were seeded into 6-well plates at a density of 1x10^5^ cells per well and cultured for 24 h. Proteins were extracted from the cells lysed in 100 μl of RIPA buffer on ice for 30 min. After centrifugation at 13,400 g at 4°C for 10 min, the supernatant was taken to determine the protein concentration using a BCA kit (Beijing Suolaibao Biotech Co., Ltd., China). 30 μg of total protein was resolved by SDS-PAGE with certain percentages of polyacrylamide separation gel corresponding to the molecular weight of the target protein, that is, 12% for TPT1, BAX, and BCL2, 10% for vimentin and p53, and 8% for all the others. The rabbit-anti-human monoclonal antibodies, anti-TPT1 (ab133568), anti-BAX (ab32503), anti-BCL2 (ab32124), anti-p53 (ab7899), and anti–N-cadherin (ab76011), were purchased from Abcam, United States. The rabbit anti-human polyclonal anti-mTOR (#2927), anti–p-mTOR (#2971), and anti-AKT (#9272) were from Cell Signalling Technology, United States. The rabbit-anti-human polyclonal anti-PI3KCA (bs-2067R) was from Beijing Biosynthesis Biotech Co., Ltd. Other anti-human monoclonal antibodies used were rabbit anti-vimentin (BF0071, Affinity Biosciences Ltd.), rabbit anti–E-cadherin (BF0219, Affinity Biosciences Ltd.), and mouse anti-GAPDH (AP0063, Nanjing Bioworld Biotech Co., Ltd.). GAPDH served as an internal reference. Membranes were then probed with HRP-conjugated goat-anti-rabbit (PMK-013–090, Pumei Biotech Co., Ltd., China) or goat-anti-mouse (EarthOx, United States) secondary antibody. Quantitative analysis was performed using ImageJ software, and the densitometry calculation was normalized against GAPDH to indicate the relative abundance of the target protein.

### RNA Extraction and Quantitative RT-PCR

An Axy Prep kit (AP-MN-MS-50, Axygen Biotech Co., Ltd., China) was used to extract total RNA from tissues or cultured cells prepared as described above for WB. The purity and concentration of RNA were measured using NanoDrop One. The reverse transcription was performed using a PrimeScrip TM RT reagent kit with a gDNA Eraser (Perfect Real Time, Takara, Japan) according to the manufacturer’s protocols.  Real-time qRT-PCR was performed using TB green Premix Rex Taq TM II (Tli RNase H Plus, Takara, Japan). The relative mRNA transcription of *TPT1* was calculated using the 2^−ΔΔCT^ method, taking the gene of human housekeeping enzyme *HPRT* as the endogenous control. Primer sequences were as follows: forward 5′-CAG​TAA​TCA​CTG​GTG​TCG​AT-3 and reverse 5′-GGA​TGT​GCT​TGA​TTT​GTT​CT-3′ for TPT1, and forward 5′-ATG​GCG​ACC​CGC​AGC​CCT-3′ and reverse 5′-CCATGAGGAATAAACACCCT-3’ for HPRT. The PCR condition was 40 cycles of 30 s 95°C, 5 s at 95°C and 30 s at 60°C.

### Biometrics

The RNA-Seq gene data of *TPT1* transcription in a cohort of 305 cervical cancer tissues were acquired from the TCGA database. The samples were divided into two groups with phenotype labels of high (*n* = 153) or low (*n* = 152) *TPT1* transcription, defined by the threshold at the median *TPT1* value. Gene Set Enrichment Analysis (GSEA3.0, UC San Diego, CA, United States) (http://software.broadinstitute.org/gsea/index.jsp) was applied to calculate and predict gene sets statistically correlating with the two phenotypes. The hallmark gene sets “h.all.v7.2.symbols.gmt” in the Molecular Signatures Database (MSigDB) on the GSEA website (http://software.broadinstitute.org/gsea/msigdb/index.jsp) were applied as reference genes. Each gene in a certain gene set was ranked by its expression difference from the cumulative running sum values, weighted using the default statistical setting of 1,000 genome permutations per analysis. An enrichment score (ES) for each gene set was recorded as the maximum deviation from zero. The *p*-value and the normalized enrichment score (NES) were used to rank the enrichment pathways of each phenotype, where a *p*-value < 0.05 calculated by Student’s *t*-test and an FDR (false discovery rates) *q*-value ≤ 0.25 were the statistical cut-offs for significant enrichment.

The online Search Tool of the Retrieval of Interacting Genes (STRING) database (http://www.string-db.org/) was used to construct the protein–protein interaction (PPI) map to calculate the strength of the association between TPT1 and key proteins in apoptosis, PI3K, and EMT pathways. Information sources of primary (experiments), predicted (databases), and textmining results in *Homo sapiens* were searched to gain an integrated overview of the pathway network. The connectivity degree was identified as network edges (lines connecting pairs of proteins) with the quantified confidence score. The minimum confidence score was set at 0.7 (high confidence).

### Statistical Analysis

SPSS 24.0 software was used to analyze the data, and correlation analysis was performed using GraphPad Prism 8.0. Two-tailed Student’s *t*-test and ANOVA (analysis of variance) were used for differential comparison between two and multiple groups, respectively. Logarithmic transformation was applied to variables that did not follow normal distribution and the nonhomogeneous variances. Numerical data conforming to the normal distribution were presented as “mean ± SD,” while the categorical data were expressed as the number of cases or the percentage rate. The chi-squared test (*χ* ^*2*^ test) and the Kruskal–Wallis rank sum test were used for comparison between two- and multi-component data, respectively. A *p-*value < 0.05 was considered statistically different.

## Results

### TPT1 Expression Is Elevated in Cervical Cancer Tissues and Cell Lines at Both Protein and mRNA Levels

**FIGURE 1:**
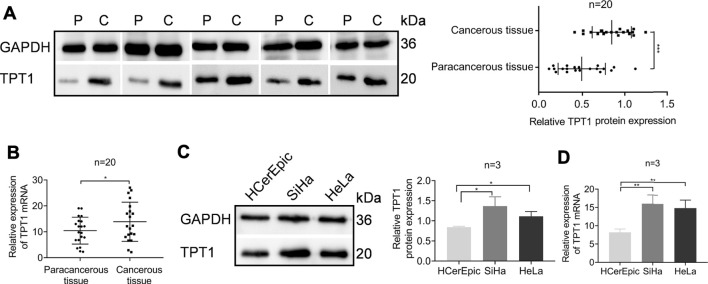
Elevated TPT1 expression in cervical cancer tissues and cells. **(A)** Protein expression of TPT1 in tissues of cervical cancer patients, assessed by WB and pairwise compared to paracancerous tissue **(left)** and quantified **(right)**. P: paracancerous tissue; C: cancerous tissue. **(B)** Relative TPT1 gene transcription in the paired cervical cancer tissues, measured by qRT-PCR. **(C)** TPT1 protein expression in normal human cervical epithelial cell line HCerEpic and cervical carcinoma cell lines SiHa and HeLa, analyzed by WB **(left)** and quantified **(right)**. **(D)** Relative TPT1 gene transcription in the cell lines, measured by qRT-PCR. WB images are representative of all samples tested. All experiments were repeated at least three times. *p < 0.05; **p < 0.01; ***p < 0.001.

Despite the ample evidence and discussion of the association between TPT1 overexpression and cancer, it is rarely studied in cervical cancer. We collected cervical cancer tissues from the local hospital to characterize the status of TPT1 expression. We found that the TPT1 protein abundance was almost doubled in the cancerous tissues compared to the paired paracancerous tissues (Figure 1A). To a lesser extent, the TPT1 mRNA transcription, revealed by qRT-PCR, was also higher (increased by 32.95%) in the cancerous than in the adjacent paracancerous tissues, despite a relatively wide overlap between the two sample groups (Figure 1B). TPT1 protein expression was also significantly higher in both human cervical carcinoma SiHa and HeLa cells than in the human normal cervical epithelial cell line HCerEpic (Figure 1C). In alignment with it, the TPT1 gene transciption was remarkably upregulated in cervical cancer cell lines (increased by 125% in SiHa cells and 111% in HeLa cells, compared to HCerEpic cells) (Figure 1D).

### The Protein Abundance of TPT1 Correlates Positively With Malignant Transformation, Lymph Node Metastasis, and the Low Differentiation Degree of Cervical Cancer

**FIGURE 2:**
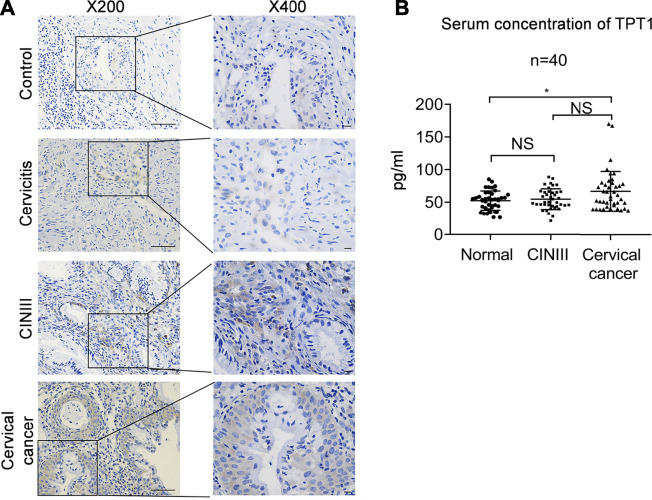
Increased abundance of TPT1 protein in the tissue and sera of cervical cancer compared to cervicitis, CINIII, and controls. **(A)** Tissue IHC staining of TPT1 protein in the control and different cervical lesions including cervicitis, CINIII, and cervical cancer. Scale bars: x200, 50 μm; x400: 10 μm. **(B)** Serum TPT1 concentration in CINIII and cervical cancer patients, compared to normal healthy controls, measured by ELISA. IHC images are representative of all samples tested. *p < 0.05; NS: statistically not significant.

Like many other common cancers, cervical cancer usually develops over a long progressive course. To get more insights into the trajectory of TPT1 expression in cervical cancer evolvement, we compared TPT1 protein levels in cervix tissues from age-matched patients with cervicitis, CINIII, and cervical cancer, by immunohistochemical staining (IHC). The HPV infection–induced cervical squamous intraepithelial neoplasia (CIN) is graded into CINI, CINII, and CINIII according to the depth of the lesion within the afflicted cervix surface. CINIII includes carcinoma *in situ* and has a high risk of developing into cancer. Whereas CIN is tightly associated with HPV infection and can involve inflammation, cervicitis is referred to as a mostly benign inflammation of the cervix that can result from a wide range of stimulations such as infection. For the control, due to the lack of cervix tissue from a healthy population, we collected cervix tissues upon hysterectomy for treatment of fibroid uteri which had been confirmed to be pathologically normal. Fibroid uteri are noncancerous myomas in the uterus and rarely include cervix lesions. A continuous increase in the TPT1 positive rate was observed in the order of control (6.7%), cervicitis (27%), CINIII (46%), and cervical cancer (69%) (Figure 2A; Table 1). TPT1 expression in circulation was also determined in CINIII and cervical cancer patients, with sera from age-matched healthy women as the normal control. Although the stepwise enhancement of TPT1 secretion from the normal baseline to CINIII and from CINIII to cancer did not reach the threshold for statistical significance, the increase was significant in cancer compared to the normal control (26.5%, *p* = 0.028) (Figure 2B; Table 2).

Among the cancerous tissues in which we quantified TPT1 abundance by WB, we further stratified the high (≥median TPT1 value) or low (<median TPT1 value) TPT1 protein expression with clinical parameters including age, FIGO (International Federation of Gynaecology and Obstetrics) stage, lymph node metastasis, and degree of cancer differentiation (Table 3). The result demonstrated that a high TPT1 protein level significantly correlated with lymph node metastasis (*p* = 0.019) and a low degree of tumor differentiation (*p* = 0.035), implying its link with EMT and cancer aggressiveness. No correlation was observed between the TPT1 status and age or the FIGO stage.

### Biometrics Analysis Reveals Differentially Expressed Gene Sets and Pathways Correlated With TPT1 Expression in Cervical Cancer

**FIGURE 3:**
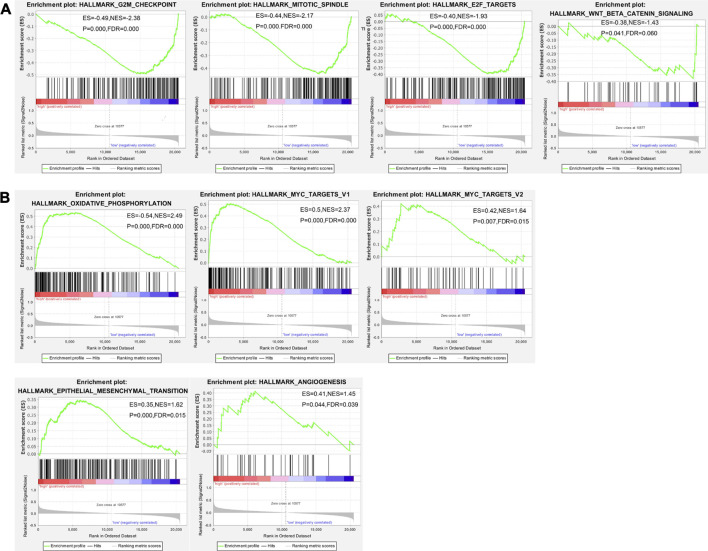
GSEA enrichment analysis of TCGA data on gene sets and pathways associated with low **(A)** or high **(B)** *TPT1* expression in cervical cancer. The horizontal bar, gradient-filled from red to blue, represents where genes in each gene set appear in the ranked list of genes. Genes on the left side (red) correlate most strongly with the phenotype. The vertical black lines represent the running enrichment scores as the projection of individual genes onto the horizontal ranked gene list. The bottom ranking matric in gray, moving from above zero (positively correlated) to below zero (negatively correlated), measures a gene’s correlation with the phenotype profile. Statistical variables of GSEA analysis are displayed in each image. ES, enrichment score for the gene set, reflecting the degree to which this gene set is over-represented at the peak (furthest from 0.0) of the entire ranked list of genes; *p-*value, statistical assessment of the significance against null distribution; NES, normalized enrichment score calculated by adjusting ES for gene set size or multiple hypothesis testing across analyzed gene sets; FDR (false discovery rate *q*-value), the estimated probability that a given NES represents a false positive finding. *p* < 0.05 and FDR < 0.25 were considered statistically significant.

The data of *TPT1* gene transcription in cervical cancer tissues were downloaded from the TCGA database and analyzed by GSEA to identify gene sets and pathways associated with differential TPT1 expression. GSEA provided us with a gene transcript profiling in certain categorical phenotype contexts, that is, high (≥median value) or low (<median value) *TPT1* transcription. Gene sets positively enriched in the low *TPT1* phenotype were genes encoding proteins involved in cell cycle arrest including G2/M checkpoint, mitotic spindle, and E2F transcription factors (Figure 3A). Genes upregulated by Wnt signaling activation through *β*-catenin accumulation were also associated with low *TPT1* levels (Figure 3A). Meanwhile, genes encoding proteins involved in oxidative phosphorylation, subgroups of genes regulated by MYC versions 1 and 2 (MYC targets V1 and V2), EMT-related genes, and upregulated genes during angiogenesis were significantly enriched in the high *TPT1* group (Figure 3B). These genes are implicated in the metabolic reprogramming, cell proliferation, transforming activity, and invasion of cervical cancer, potentially in concert with TPT1 activity.

**FIGURE 4:**
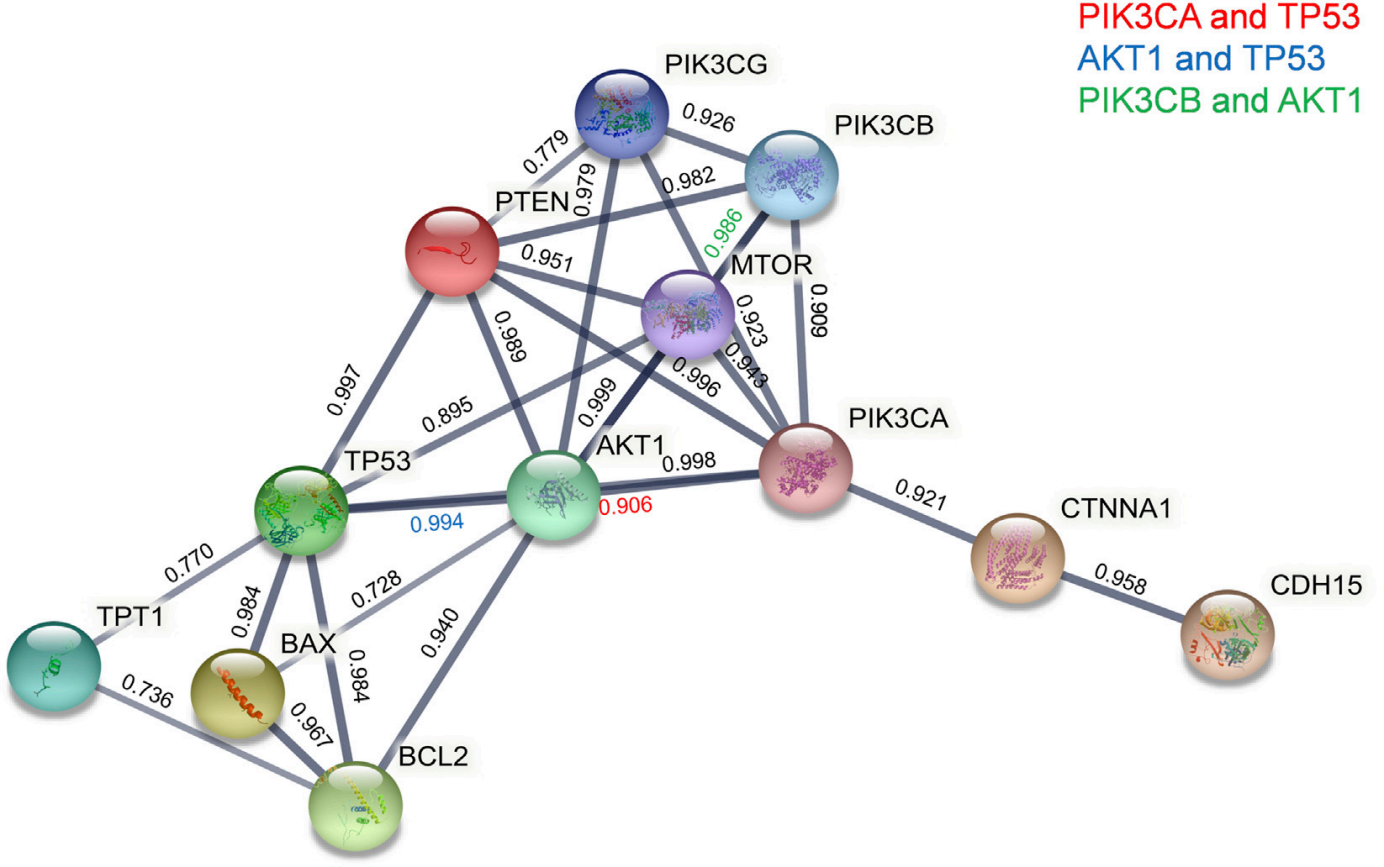
STRING PPI network analyses of functional connections of TPT1 with key proteins of PI3K/AKT/mTOR signaling, apoptosis, and EMT pathways. Line thickness indicates the strength of data support, and circles in different colors represent individual proteins with the abbreviated name. The number above each line is the combined association score that indicates a probability of the existence of a real functional association between assigned proteins, calculated from online sources of textmining, experiments, and databases. The threshold of the confidence score was set at 0.7 (high).

We next sought an understanding of how the TPT1 protein is functionally involved in pathways of apoptosis, proliferation, and EMT, the most frequently dysregulated pathways during cervical cancer development. A map of protein–protein interactions (PPIs) between TPT1 and key factors regulating apoptosis, PI3K cascade, and intercellular adhesion was created by mining the Search Tool for the Retrieval of Interacting Genes (STRING) to predict their functional cooperation in biological events (Figure 4). The map shows that TPT1 directly interacts with TP53 (a combined association score of 0.770) and BCL2 (a combined association score of 0.736). The proapoptotic protein BAX and the antiapoptotic BCL2 are both transcriptional targets of p53. The interactions among them suggest that abnormal TPT1 activity could impair the cascade of apoptosis, *via* dysregulation of p53 and BCL2. With a very high combined association score above 0.9, TP53 and BCL2 lead the interaction network to a cluster of proteins related to PI3K/AKT/mTOR signal transduction (PTEN, AKT1, mTOR. PI3CA, PI3CB, and PI3CG), which is critical in bioprocesses of cell cycle and proliferation. Members of the catenin family (CTNNA1) and the cadherin superfamily (CDH15) are also linked with the cluster at very high association scores—all above 0.9. These factors play roles in cell adhesion, thereby being involved in cancer metastasis.

### Key Factors in Pathways Related to TPT1 Regulation and Function Are Altered in Cervical Cancer Tissues and Cells

**FIGURE 5:**
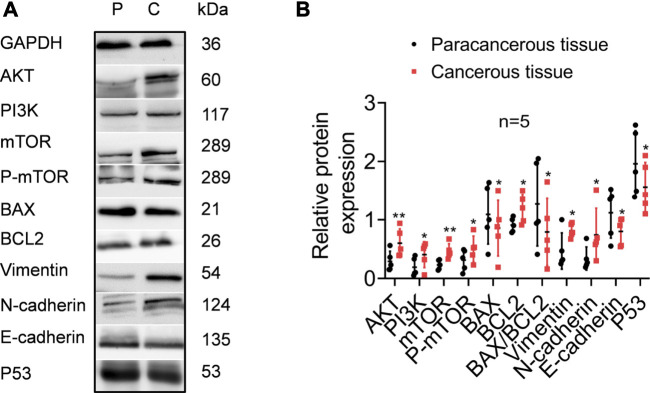
Identification of key proteins involved in PI3K/AKT/mTOR pathway over-activation, apoptosis suppression, EMT, and p53 degradation in cancerous tissues of cervical cancer patients, in comparison with the paracancerous tissues. **(A)** WB images that are representative of all samples tested. **(B)** Densitometry quantification of the WB results from the experiment performed in A. The ratio of BAX:BCL2 is calculated to highlight the apoptosis activity. *p < 0.05. **p < 0.01. *** p < 0.001.

**FIGURE 6:**
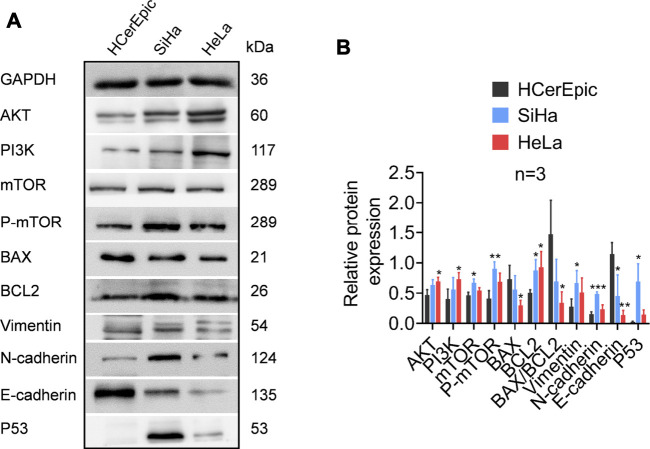
Identification of proteins involved in PI3K/AKT/mTOR pathway over-activation, apoptosis suppression, and EMT in cervical carcinoma cell lines SiHa and HeLa, in comparison with the non-malignant cervical epithelial cell line HCerEpic. **(A)** WB images that are representative of all replicates of the experiment. **(B)** Densitometry quantification of the WB results from the experiment performed in A. The ratio of BAX:BCL2 is calculated to highlight the apoptosis activity. *p < 0.05. **p < 0.01. ***p < 0.001.

TPT1 participates in the precise and accurate regulation of various bioprocesses. Although in this work we could not directly examine its mechanistic role in cervical cancer development, we compared the activities of the PI3K/AKT/mTOR pathway, apoptosis, and EMT markers between cancerous and paracancerous tissues of cervical cancer patients (Figure 5). According to reports in the literature and our GSEA/STRING data, these factors are closely linked with TPT1 regulation and functions. As anticipated, in comparison to the paracancerous control, the cancerous tissues had significantly higher protein expression of PI3K, AKT, mTOR, and p-mTOR, accompanied by a lower BAX:BCL2 ratio (decreased by 37.80%) that indicated a status with favored growth signaling and restrained apoptosis. Moreover, the expression switch from E-cadherin to N-cadherin in cancer tissues suggested the activation of EMT, along with the overexpression of vimentin, an important EMT marker. Meanwhile, p53 expression was significantly reduced (Figures 5A,B). It is known that p53 and TPT1 form an antagonizing regulatory circuit where p53 directly represses TPT1 transcription and TPT1 promotes p53 degradation (Amson et al., 2011). Similar signatures of PI3K/AKT/mTOR over-activation, blocking of apoptosis, and EMT activity were detected in both SiHa and HeLa cells, in comparison to HCerEpic cells, except that the change in vimentin was not evident (Figures 6A,B). Besides, the level of p53 in the cancer cell lines did not display an inversed relationship with TPT1 expression, as we might have expected. The causative relationships between these pathways with TPT1 overexpression in cervical cancer warrant verification.

## Discussion

TPT1 mRNA and viral particles share a similar structure and trigger an innate immune reaction in a similar way (Amson et al., 2013). The dual function of TPT1 in defense against infection and promoting cell survival, two events deterministic for the cellular fate in cervical cancer, underlines its importance in cervical cancer development. However, to the best of our knowledge, the present study is the first to report its relevance to cervical cancer progression in patients. We also sought to address the differential expression of TPT1 in noncancerous cervical lesions including cervicitis and pre-cancerous CINIII neoplasia. The data suggested a continuum of increased TPT1 expression leading up to malignant transformation. Bearing in mind that TPT1 might execute different functions in the context of benign cervical inflammation, its steady increase through cancer development suggests that TPT1 is an important signature manifesting itself long before cancer diagnosis. The finding that high expression of TPT1 significantly correlated with both lymph node metastasis and cancer cell differentiation implies that it may promote cancer progression. However, this was not echoed in FIGO staging, which could be explained by the small number of sample cases and the fact that no samples at FIGO stages III and IV were available.

The invasion of the HPV oncogenes is the major etiological cause of cervical cancer and the precursor epithelial lesions. Malignant transformation of cervical cancer ensues from failure in the defense against the virus infection, manifesting as interrupted cellular machinery, including DNA replication, inefficient immune response, chronic inflammation (Boccardo et al., 2010), disruption of p53 expression and function (Ruttkay-Nedecky et al., 2013), and abnormal PI3K/AKT/mTOR signaling (Zhang et al., 2015; Bossler et al., 2019). The close relationship between TPT1 and HPV infection is also shown in a proteomics profiling of lung A549 cells stably infected by HPV16E6/E7, where the cyto-protective TPT1 was remarkably upregulated (Ciotti et al., 2009). For the small number of HPV-negative cervical cancers, over-activation of the PI3K/AKT/mTOR pathway or mutations of its regulatory factors, such as PTEN, EGFR, and HER2, accounted for the virus-independent etiology of carcinogenesis (Tjalma, 2018).

In our study, biometrics data mining substantially compensated for the limitation of the sample size and the scarcity of experimental reference. Both GSEA enrichment analysis and STRING mapping provided information that has resonance with the near-ubiquitous engagement of TPT1 in tumorigenesis found in other cancers (Amson et al., 2013; Lee et al., 2020), including growth promotion *via* PI3K signaling, antiapoptotic activities, EMT, and the reciprocal interaction with p53. Although we could not clarify whether the increased TPT1 was a phenotype secondary to other alterations in cervical cancer, it is plausible to speculate that TPT1 was proactively involved in the examined pathways. Unlike the cervical cancer cell lines, where a dramatic upregulation of TPT1 at both mRNA and protein levels was detected, the relative TPT1 gene transcription in about half of the cancerous tissue samples did not distinguish themselves from their paracancerous controls. Nevertheless, the mean value still met the statistical requirement of significance. Regardless of the small sample size, it might imply a predominance of post-transcriptional tuning of TPT1 in the cervical cancer niche. However, before further evidence becomes available, we ought not to rush into any speculation.

We also found that the serum TPT1 concentrations had a tendency to increase in CINIII and cancer patients and was significantly higher in cancer patients than in the healthy population. Monitoring the alteration of the TPT1 protein at the systemic level in cervical cancer is especially meaningful as it could considerably alleviate the diagnostic burden and may serve as a convenient biomarker to assist early detection of cancer or treatment evaluation.

The multiplicity of TPT1 function in the pathway network, evident in most common cancers and tentatively inferred by our data, offers a great impetus to investigate its therapeutic potential in cervical cancer management. A combined mechanism that evokes an intracellular reprogramming to restore the antitumor defense system was proposed (Tuynder et al., 2002). Moreover, depletion of TPT1 in HeLa cells or mice not only promoted apoptosis by BCL2 inhibition but enhanced the overall autophagy flux (Bae et al., 2017), two attractive strategies against cancer. Interestingly, some existing drugs are emerging as efficient anticancer agents *via* TPT1 inhibition or knockdown. For example, the antidepressant Sertraline and the antipsychotic drug Thioridazine were tested successfully *ex vivo* on primary AMT cells, reducing cell viability by inactivating TPT1 (RaP et al., 2013). Similar treatment on the colon cancer cell line HCT116 retrieved wild-type p53 function and provoked apoptosis in the cells (Amson et al., 2011). Inhibition of TPT1 by binding an anti-malaria drug, Dihydroartemisinin (DHA), to its phosphorylated form in breast cancer cell lines reduced cell growth and induced apoptosis (Lucibello et al., 2015).

In conclusion, we demonstrate a positive correlation between TPT1 expression and cervical cancer progression. Studies with larger cohorts and cellular and animal models are intensely ongoing, conducted by our team, to further consolidate the results and unravel the underlying mechanisms. In particular, evaluation of the significance of TPT1 delivered *via* circulation would hugely benefit cervical cancer diagnosis. Three merits of bringing TPT1 onboard in the battle against cervical cancer are in sight—opening an earlier window for cervical cancer intervention, circumvention of the obstacle of tissue sampling, and a prompt establishment of a tractable therapy option using current anti-TPT1 drugs.

## Data Availability

The datasets presented in this study can be found in online repositories. The names of the repository/repositories and accession number(s) can be found in the article/Supplementary Material.
